# *N*,*N*,*N*′,*N*′-Tetra­methyl­phthalamide

**DOI:** 10.1107/S1600536812035167

**Published:** 2012-08-15

**Authors:** Adel Hamada, Yamina Boudinar, Adel Beghidja, Mehdi Boutebdja

**Affiliations:** a Département des Sciences Fondamentales, Faculté des Sciences, Université du 20 Août 1955 – Skikda, Route d’El-Hadaïk, BP 26, 21000 Skikda, Algeria; b Unité de Recherche de Chimie de l’Environnement et Moléculaire Structurale (CHEMS), Faculté des Sciences Exactes, Département de Chimie, Université-Mentouri, 25000 Constantine, Algeria

## Abstract

The title compound, C_12_H_16_N_2_O_2_, crystallized from toluene with two independent mol­ecules in the asymmetric unit. The dihedral angles between the amide groups and the benzene ring are 60.87 (11) and 54.08 (11)° in one independent molecule and 60.13 (11) and 64.64 (11) in the other. The crystal structure features weak C—H⋯O hydrogen bonds and C—H⋯π inter­actions.

## Related literature

For related structures, see: Altamura *et al.* (2005 ▶); Anderson *et al.* (2004 ▶); Clayden *et al.* (2001 ▶); Comins *et al.* (1998 ▶); Sakamoto *et al.* (2004 ▶). 
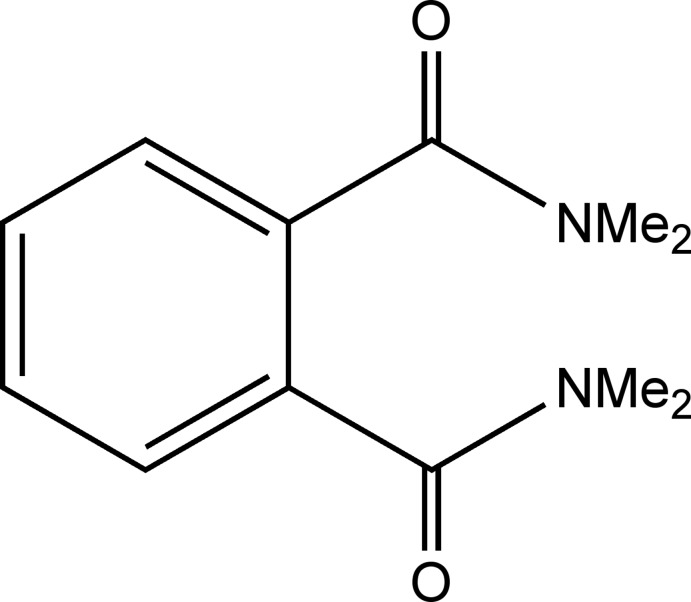


## Experimental

### 

#### Crystal data

C_12_H_16_N_2_O_2_
*M* *_r_* = 220.27
Monoclinic, 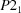

*a* = 6.7337 (6) Å
*b* = 18.1230 (14) Å
*c* = 9.8216 (8) Å
β = 104.918 (3)°
*V* = 1158.18 (17) Å^3^
*Z* = 4
Mo *K*α radiation
μ = 0.09 mm^−1^
*T* = 100 K
0.56 × 0.52 × 0.33 mm

#### Data collection

Bruker APEXII diffractometer
Absorption correction: multi-scan (*SADABS*; Sheldrick, 2002 ▶) *T*_min_ = 0.925, *T*_max_ = 0.972
8205 measured reflections
2739 independent reflections
2414 reflections with *I* > 2σ(*I*)
*R*_int_ = 0.045

#### Refinement

*R*[*F* ^2^ > 2σ(*F* ^2^)] = 0.049
*wR*(*F* ^2^) = 0.124
*S* = 1.06
2739 reflections
297 parameters
1 restraint
H-atom parameters constrained
Δρ_max_ = 0.32 e Å^−3^
Δρ_min_ = −0.34 e Å^−3^

### 

Data collection: *APEX2* (Bruker, 2006 ▶); cell refinement: *SAINT* (Bruker, 2006 ▶); data reduction: *SAINT*; program(s) used to solve structure: *SHELXS97* (Sheldrick, 2008 ▶); program(s) used to refine structure: *SHELXL97* (Sheldrick, 2008 ▶); molecular graphics: *ATOMS* (Dowty, 1995 ▶); software used to prepare material for publication: *WinGX* (Farrugia, 1999 ▶).

## Supplementary Material

Crystal structure: contains datablock(s) global, I. DOI: 10.1107/S1600536812035167/bq2372sup1.cif

Structure factors: contains datablock(s) I. DOI: 10.1107/S1600536812035167/bq2372Isup2.hkl

Supplementary material file. DOI: 10.1107/S1600536812035167/bq2372Isup3.cml

Additional supplementary materials:  crystallographic information; 3D view; checkCIF report

## Figures and Tables

**Table 1:** Hydrogen-bond geometry (Å, °) *Cg*1 is the centroid of the C2–C7 ring.

*D*—H⋯*A*	*D*—H	H⋯*A*	*D*⋯*A*	*D*—H⋯*A*
C3—H3⋯O3^i^	0.93	2.60	3.518 (3)	170
C5—H5⋯O4^ii^	0.93	2.58	3.141 (4)	119
C6—H6⋯O4^ii^	0.93	2.51	3.105 (4)	122
C18—H18⋯O2^iii^	0.93	2.49	3.329 (3)	150
C24—H24*A*⋯O2^iii^	0.96	2.56	3.224 (4)	127
C16—H16⋯*Cg*1	0.93	2.97	3.810 (4)	124

Symmetry codes: (i) 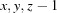; (ii) 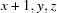; (iii) 
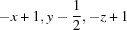
.

**Fig. 1.**
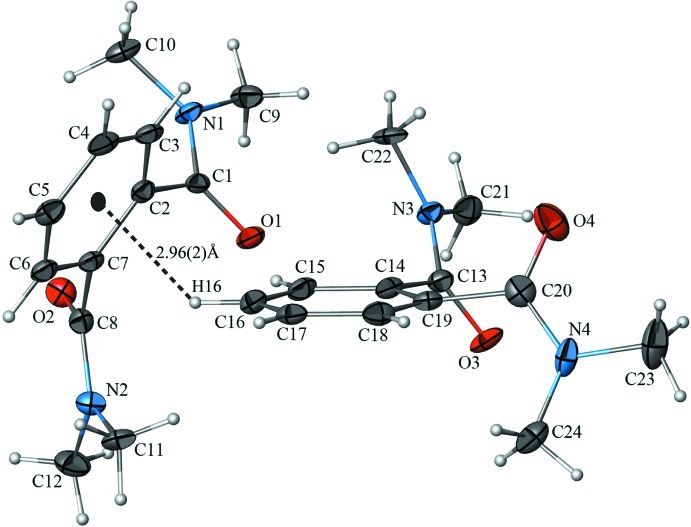
View of the two molecules in the asymmetric unit of (I) showing the numbering schemes; displacement ellipsoids are drawn at the 50% probability level.

**Fig. 2.**
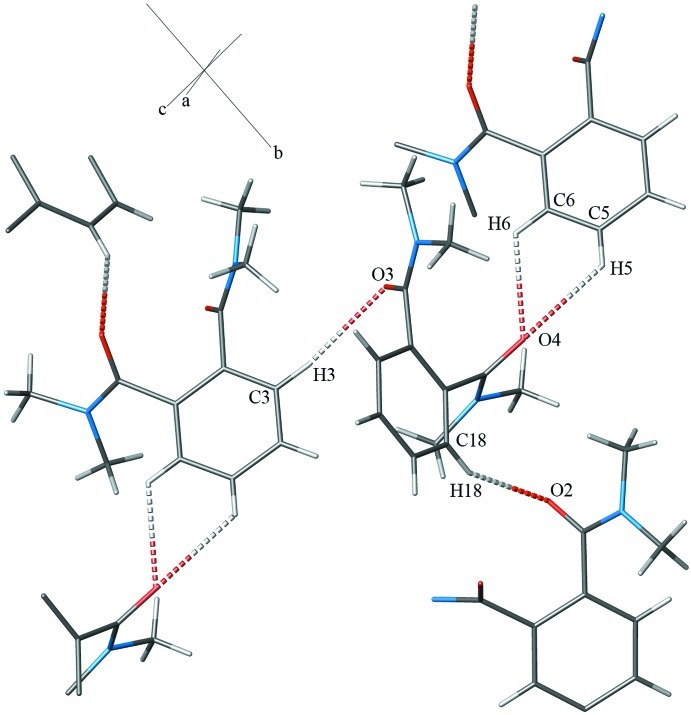
Partial view of the crystal structure of (I) showing the non standard hydrogen bonds.

## supplementary crystallographic information

### Comment

The molecular structure of (I) is composed of a two crystallographically independent molecules (IA and IB). A displacement ellipsoid plot of the two independent molecules of (I) is shown in Fig. 1. Bond lengths and angles in (I) are normal, and values for the two independent molecules agree with each other. The crystal structure of (I) is stabilized by intermolecular weak hydrogen-bonds type C—H···O (Fig. 2) and C—H··· π interactions, the latter interaction is observed between centroid of benzene ring (C2—C7) (IA) and hydrogen atom H16 of benzene ring (C14—C19) of the adjacent molecule (IB), with a *Cg*···H16 distance of 2.96 (2) Å (Fig. 1), resulting in the formation of infinite three-dimensional network reinforcing a cohesion of structure. Hydrogen-bonding parameters are listed in Table 1.

### Experimental

To a suspension of phthalic acid (1 g, 6.02 mmol) in anhydrous toluene (10 ml) trimethlphosphine (6.02 mmol) was added and the mixture kept under reflux for 45 min. The resulting cloudy solution was allowed to cool to room temperature and a saturated aqueous solution of NaHCO3 (5 ml) was added. The layers were separated and the aqueous phase was extracted with methylene chloride (4.5 ml). The organic solutions were combined together, dried over anhydrous MgSO_4_ and concentrated to dryness, obtaining a white solid. Colorless single crystals suitable for X-ray diffraction analysis were obtained by dissolving the corresponding compound in toluene solution and letting it for slow evaporation at room temperature (Yield: 1.30 g, 92%).

### Refinement

All non-hydrogen atoms were refined with anisotropic atomic displacement parameters. All H atoms were placed at calculated positions and treated as riding on their parent atoms with C—H = 0.93–0.96 Å, and *U*_iso_ (H) = 1.5Ueq(C) for methyl H atoms and 1.2Ueq(C) for the others. The absolute structure parameter is meaningless because the compound is a weak anomalous scatterer. So, the absolute structure parameter is removed from the CIF.

### Crystal data

**Table:**

C_12_H_16_N_2_O_2_	*F*(000) = 472
*M**_r_* = 220.27	*D*_x_ = 1.263 Mg m^−^^3^
Monoclinic, *P*2_1_	Melting point: 118 K
Hall symbol: P 2yb	Mo *K*α radiation, λ = 0.71073 Å
*a* = 6.7337 (6) Å	Cell parameters from 2761 reflections
*b* = 18.1230 (14) Å	θ = 2.4–26.8°
*c* = 9.8216 (8) Å	µ = 0.09 mm^−^^1^
β = 104.918 (3)°	*T* = 100 K
*V* = 1158.18 (17) Å^3^	Needles, colourless
*Z* = 4	0.56 × 0.52 × 0.33 mm

### Data collection

**Table:**

Bruker APEXII diffractometer	2739 independent reflections
Graphite monochromator	2414 reflections with *I* > 2σ(*I*)
Detector resolution: 18.4 pixels mm^-1^	*R*_int_ = 0.045
CCD rotation images, thin slices scans	θ_max_ = 27.5°, θ_min_ = 2.4°
Absorption correction: multi-scan (*SADABS*; Sheldrick, 2002)	*h* = −8→8
*T*_min_ = 0.925, *T*_max_ = 0.972	*k* = −23→20
8205 measured reflections	*l* = −11→12

### Refinement

**Table:**

Refinement on *F*^2^	Primary atom site location: structure-invariant direct methods
Least-squares matrix: full	Secondary atom site location: difference Fourier map
*R*[*F*^2^ > 2σ(*F*^2^)] = 0.049	Hydrogen site location: inferred from neighbouring sites
*wR*(*F*^2^) = 0.124	H-atom parameters constrained
*S* = 1.06	*w* = 1/[σ^2^(*F*_o_^2^) + (0.0785*P*)^2^] where *P* = (*F*_o_^2^ + 2*F*_c_^2^)/3
2739 reflections	(Δ/σ)_max_ < 0.001
297 parameters	Δρ_max_ = 0.32 e Å^−^^3^
1 restraint	Δρ_min_ = −0.34 e Å^−^^3^

### Special details

**Table:**

Geometry. Bond distances, angles *etc*. have been calculated using the rounded fractional coordinates. All su's are estimated from the variances of the (full) variance-covariance matrix. The cell e.s.d.'s are taken into account in the estimation of distances, angles and torsion angles
Refinement. Refinement on *F*^2^ for ALL reflections except those flagged by the user for potential systematic errors. Weighted *R*-factors *wR* and all goodnesses of fit *S* are based on *F*^2^, conventional *R*-factors *R* are based on *F*, with *F* set to zero for negative *F*^2^. The observed criterion of *F*^2^ > σ(*F*^2^) is used only for calculating -*R*-factor-obs *etc*. and is not relevant to the choice of reflections for refinement. *R*-factors based on *F*^2^ are statistically about twice as large as those based on *F*, and *R*-factors based on ALL data will be even larger.

### Fractional atomic coordinates and isotropic or equivalent isotropic displacement parameters (Å^2^)

**Table:**

	*x*	*y*	*z*	*U*_iso_*/*U*_eq_	
O1	0.7861 (3)	0.24084 (11)	−0.0065 (2)	0.0216 (6)	
O2	0.8041 (3)	0.28421 (11)	0.3142 (2)	0.0235 (6)	
N1	0.4493 (3)	0.26282 (13)	−0.0175 (2)	0.0195 (7)	
N2	1.0989 (4)	0.21659 (13)	0.3653 (2)	0.0220 (7)	
C1	0.6269 (4)	0.22638 (15)	0.0296 (3)	0.0171 (7)	
C2	0.6276 (4)	0.16096 (14)	0.1249 (3)	0.0168 (7)	
C3	0.5027 (4)	0.10057 (15)	0.0751 (3)	0.0189 (7)	
C4	0.5113 (4)	0.03795 (16)	0.1583 (3)	0.0235 (8)	
C5	0.6447 (4)	0.03548 (15)	0.2915 (3)	0.0211 (8)	
C6	0.7738 (4)	0.09499 (15)	0.3414 (3)	0.0200 (7)	
C7	0.7675 (4)	0.15789 (14)	0.2577 (3)	0.0171 (7)	
C8	0.8934 (4)	0.22495 (16)	0.3135 (3)	0.0189 (7)	
C9	0.4327 (5)	0.32006 (16)	−0.1231 (3)	0.0244 (8)	
C10	0.2644 (4)	0.25242 (16)	0.0331 (3)	0.0232 (8)	
C11	1.2112 (4)	0.14818 (17)	0.3610 (3)	0.0261 (8)	
C12	1.2221 (5)	0.27854 (19)	0.4338 (3)	0.0312 (9)	
O3	0.1819 (3)	0.12996 (12)	0.7353 (2)	0.0259 (6)	
O4	−0.0391 (4)	−0.02611 (13)	0.5606 (2)	0.0358 (7)	
N3	−0.1580 (3)	0.14947 (12)	0.7148 (2)	0.0186 (6)	
N4	0.2973 (4)	−0.05367 (13)	0.6492 (3)	0.0258 (8)	
C13	0.0169 (4)	0.11065 (15)	0.7583 (3)	0.0188 (7)	
C14	0.0152 (4)	0.04326 (15)	0.8474 (3)	0.0180 (7)	
C15	−0.0313 (4)	0.05018 (16)	0.9770 (3)	0.0209 (8)	
C16	−0.0171 (5)	−0.01003 (17)	1.0661 (3)	0.0224 (8)	
C17	0.0435 (4)	−0.07820 (16)	1.0259 (3)	0.0209 (8)	
C18	0.0923 (4)	−0.08572 (14)	0.8968 (3)	0.0206 (7)	
C19	0.0795 (4)	−0.02558 (15)	0.8073 (3)	0.0189 (7)	
C20	0.1098 (5)	−0.03514 (15)	0.6626 (3)	0.0235 (8)	
C21	−0.1553 (5)	0.22148 (16)	0.6495 (3)	0.0244 (8)	
C22	−0.3615 (4)	0.12250 (17)	0.7172 (3)	0.0243 (8)	
C23	0.3187 (7)	−0.0733 (2)	0.5096 (4)	0.0426 (13)	
C24	0.4731 (5)	−0.06937 (18)	0.7671 (3)	0.0292 (9)	
H3	0.41290	0.10210	−0.01430	0.0230*	
H4	0.42730	−0.00230	0.12440	0.0280*	
H5	0.64820	−0.00600	0.34780	0.0250*	
H6	0.86440	0.09290	0.43050	0.0240*	
H9A	0.56090	0.32440	−0.14820	0.0370*	
H9B	0.40060	0.36620	−0.08570	0.0370*	
H9C	0.32570	0.30750	−0.20520	0.0370*	
H10A	0.29320	0.21810	0.11010	0.0350*	
H10B	0.15540	0.23350	−0.04200	0.0350*	
H10C	0.22350	0.29890	0.06420	0.0350*	
H11A	1.13770	0.11860	0.28320	0.0390*	
H11B	1.22450	0.12150	0.44730	0.0390*	
H11C	1.34530	0.15940	0.34950	0.0390*	
H12A	1.13530	0.32080	0.43170	0.0470*	
H12B	1.32500	0.28980	0.38510	0.0470*	
H12C	1.28750	0.26590	0.52980	0.0470*	
H15	−0.07230	0.09570	1.00410	0.0250*	
H16	−0.04820	−0.00480	1.15250	0.0270*	
H17	0.05160	−0.11880	1.08500	0.0250*	
H18	0.13380	−0.13130	0.87050	0.0250*	
H21A	−0.01970	0.23140	0.63960	0.0370*	
H21B	−0.19320	0.25870	0.70760	0.0370*	
H21C	−0.25120	0.22170	0.55830	0.0370*	
H22A	−0.34750	0.07910	0.77490	0.0360*	
H22B	−0.43820	0.11070	0.62300	0.0360*	
H22C	−0.43260	0.16000	0.75520	0.0360*	
H23A	0.22120	−0.04580	0.43950	0.0640*	
H23B	0.29370	−0.12510	0.49400	0.0640*	
H23C	0.45540	−0.06180	0.50360	0.0640*	
H24A	0.47420	−0.12090	0.79030	0.0440*	
H24B	0.46360	−0.04050	0.84720	0.0440*	
H24C	0.59760	−0.05710	0.74170	0.0440*	

### Atomic displacement parameters (Å^2^)

**Table:**

	*U*^11^	*U*^22^	*U*^33^	*U*^12^	*U*^13^	*U*^23^
O1	0.0140 (10)	0.0250 (10)	0.0271 (10)	−0.0016 (8)	0.0076 (8)	0.0017 (8)
O2	0.0234 (11)	0.0206 (9)	0.0257 (10)	0.0012 (9)	0.0047 (9)	−0.0004 (8)
N1	0.0116 (11)	0.0236 (12)	0.0242 (11)	0.0015 (10)	0.0063 (9)	0.0041 (9)
N2	0.0166 (12)	0.0240 (11)	0.0235 (11)	−0.0029 (10)	0.0019 (10)	−0.0011 (9)
C1	0.0128 (12)	0.0197 (12)	0.0185 (12)	−0.0004 (11)	0.0033 (10)	−0.0047 (10)
C2	0.0144 (12)	0.0182 (11)	0.0193 (12)	0.0025 (10)	0.0069 (10)	−0.0008 (10)
C3	0.0087 (12)	0.0237 (12)	0.0245 (13)	0.0005 (11)	0.0044 (10)	−0.0019 (11)
C4	0.0134 (13)	0.0222 (13)	0.0365 (15)	−0.0026 (11)	0.0094 (12)	−0.0036 (12)
C5	0.0181 (14)	0.0188 (12)	0.0290 (14)	0.0027 (11)	0.0106 (12)	0.0048 (11)
C6	0.0149 (13)	0.0226 (12)	0.0233 (13)	0.0029 (11)	0.0066 (11)	−0.0009 (10)
C7	0.0117 (12)	0.0188 (12)	0.0215 (12)	0.0017 (11)	0.0057 (10)	−0.0017 (10)
C8	0.0178 (13)	0.0228 (12)	0.0169 (12)	0.0005 (12)	0.0058 (10)	0.0026 (10)
C9	0.0179 (14)	0.0258 (13)	0.0273 (14)	0.0004 (12)	0.0020 (12)	0.0050 (11)
C10	0.0127 (13)	0.0242 (13)	0.0338 (15)	0.0013 (11)	0.0082 (12)	0.0014 (12)
C11	0.0122 (13)	0.0331 (15)	0.0310 (15)	0.0020 (13)	0.0017 (12)	0.0005 (12)
C12	0.0271 (17)	0.0319 (15)	0.0297 (15)	−0.0084 (15)	−0.0014 (13)	−0.0054 (13)
O3	0.0141 (10)	0.0294 (10)	0.0368 (11)	−0.0012 (9)	0.0111 (9)	0.0060 (9)
O4	0.0446 (15)	0.0339 (11)	0.0247 (11)	0.0143 (12)	0.0015 (10)	0.0000 (9)
N3	0.0120 (11)	0.0202 (11)	0.0231 (11)	0.0032 (9)	0.0036 (9)	0.0016 (9)
N4	0.0330 (15)	0.0220 (11)	0.0273 (13)	0.0061 (11)	0.0165 (12)	0.0022 (9)
C13	0.0145 (13)	0.0218 (12)	0.0203 (12)	0.0007 (11)	0.0047 (10)	−0.0033 (10)
C14	0.0092 (12)	0.0189 (12)	0.0242 (13)	0.0001 (10)	0.0010 (10)	−0.0006 (10)
C15	0.0127 (13)	0.0236 (12)	0.0265 (14)	0.0004 (11)	0.0052 (11)	−0.0040 (11)
C16	0.0159 (14)	0.0285 (14)	0.0241 (13)	−0.0028 (12)	0.0076 (12)	−0.0013 (11)
C17	0.0112 (13)	0.0239 (13)	0.0261 (14)	−0.0042 (11)	0.0020 (11)	0.0025 (11)
C18	0.0166 (13)	0.0177 (12)	0.0259 (13)	−0.0007 (11)	0.0025 (11)	−0.0029 (10)
C19	0.0109 (13)	0.0204 (12)	0.0239 (13)	−0.0003 (11)	0.0019 (10)	−0.0011 (10)
C20	0.0293 (17)	0.0163 (12)	0.0245 (14)	0.0042 (12)	0.0060 (13)	0.0017 (10)
C21	0.0225 (15)	0.0248 (13)	0.0277 (14)	0.0064 (13)	0.0098 (12)	0.0050 (12)
C22	0.0084 (13)	0.0300 (14)	0.0321 (15)	0.0020 (12)	0.0008 (11)	0.0025 (12)
C23	0.064 (3)	0.0364 (17)	0.0380 (19)	0.0132 (19)	0.0323 (19)	0.0050 (15)
C24	0.0218 (16)	0.0261 (14)	0.0431 (17)	−0.0013 (13)	0.0145 (14)	−0.0019 (13)

### Geometric parameters (Å, º)

**Table:**

O1—C1	1.241 (3)	C10—H10C	0.9600
O2—C8	1.232 (4)	C10—H10B	0.9600
O3—C13	1.240 (3)	C11—H11B	0.9600
O4—C20	1.232 (4)	C11—H11C	0.9600
N1—C10	1.467 (3)	C11—H11A	0.9600
N1—C1	1.340 (4)	C12—H12B	0.9600
N1—C9	1.451 (4)	C12—H12C	0.9600
N2—C11	1.459 (4)	C12—H12A	0.9600
N2—C8	1.354 (4)	C13—C14	1.504 (4)
N2—C12	1.454 (4)	C14—C15	1.393 (4)
N3—C13	1.344 (3)	C14—C19	1.410 (4)
N3—C22	1.461 (4)	C15—C16	1.387 (4)
N3—C21	1.456 (4)	C16—C17	1.390 (4)
N4—C23	1.459 (5)	C17—C18	1.396 (4)
N4—C24	1.455 (4)	C18—C19	1.389 (4)
N4—C20	1.345 (4)	C19—C20	1.497 (4)
C1—C2	1.510 (4)	C15—H15	0.9300
C2—C7	1.400 (4)	C16—H16	0.9300
C2—C3	1.390 (4)	C17—H17	0.9300
C3—C4	1.391 (4)	C18—H18	0.9300
C4—C5	1.384 (4)	C21—H21A	0.9600
C5—C6	1.393 (4)	C21—H21B	0.9600
C6—C7	1.400 (4)	C21—H21C	0.9600
C7—C8	1.502 (4)	C22—H22A	0.9600
C3—H3	0.9300	C22—H22B	0.9600
C4—H4	0.9300	C22—H22C	0.9600
C5—H5	0.9300	C23—H23A	0.9600
C6—H6	0.9300	C23—H23B	0.9600
C9—H9A	0.9600	C23—H23C	0.9600
C9—H9B	0.9600	C24—H24A	0.9600
C9—H9C	0.9600	C24—H24B	0.9600
C10—H10A	0.9600	C24—H24C	0.9600
			
O1···O2	3.218 (3)	C16···H4^v^	2.9000
O1···C8	3.052 (3)	C17···H4^v^	2.8700
O1···C10^i^	3.150 (3)	C17···H10C^x^	2.8600
O1···C22^ii^	3.400 (4)	C18···H24B	2.7900
O1···C18^iii^	3.355 (3)	C18···H10C^x^	3.0800
O2···C24^iii^	3.224 (4)	C18···H24A	3.0800
O2···C1	2.936 (3)	C19···H24B	2.5300
O2···C20^iii^	3.322 (3)	C22···H15	3.0200
O2···N4^iii^	3.059 (3)	C24···H18	2.9500
O2···C23^iii^	3.329 (4)	C24···H22A^i^	2.9400
O2···C18^iii^	3.329 (3)	H3···C14^vii^	2.8800
O2···O1	3.218 (3)	H3···O3^vii^	2.6000
O3···C20	3.085 (3)	H3···N1	2.9200
O3···C22^i^	3.128 (3)	H3···C10	2.9800
O4···C13	3.111 (4)	H3···C13^vii^	3.0100
O4···C6^iv^	3.105 (4)	H4···C16^vii^	2.9000
O4···C5^iv^	3.141 (4)	H4···C17^vii^	2.8700
O1···H21B^ii^	2.8700	H5···O4^i^	2.5800
O1···H22C^ii^	2.8300	H5···H23C	2.4600
O1···H9A	2.3300	H6···C11	2.7800
O1···H18^iii^	2.6600	H6···H11B	2.4400
O1···H10B^i^	2.6000	H6···O4^i^	2.5100
O1···H15^ii^	2.7900	H6···N2	2.9100
O2···H12A	2.3300	H9A···O1	2.3300
O2···H24A^iii^	2.5600	H9B···H10C	2.4500
O2···H18^iii^	2.4900	H9C···H10B	2.5700
O2···H21C^i^	2.7600	H10A···H11C^iv^	2.5200
O2···H23B^iii^	2.7100	H10A···C11^iv^	2.9500
O3···H11B^iv^	2.9200	H10A···C2	2.4500
O3···H21A	2.3300	H10A···C3	2.6300
O3···H3^v^	2.6000	H10B···H9C	2.5700
O3···H22C^i^	2.6100	H10B···C13^vii^	2.9500
O4···H6^iv^	2.5100	H10B···O1^iv^	2.6000
O4···H23A	2.3800	H10C···H9B	2.4500
O4···H5^iv^	2.5800	H10C···C17^xi^	2.8600
O4···H12A^vi^	2.8500	H10C···C18^xi^	3.0800
N4···O2^vi^	3.059 (3)	H10C···H17^xi^	2.5300
N1···H3	2.9200	H11A···C6	2.6900
N2···H6	2.9100	H11A···C7	2.5400
N3···H15	2.9200	H11B···O3^i^	2.9200
C1···O2	2.936 (3)	H11B···C6	2.9800
C3···C15^vii^	3.593 (4)	H11B···H6	2.4400
C3···C10	3.159 (4)	H11B···H22B^ix^	2.4800
C4···C16^vii^	3.546 (4)	H11C···H10A^i^	2.5200
C5···O4^i^	3.141 (4)	H11C···H12B	2.4000
C6···C11	3.058 (4)	H12A···O2	2.3300
C6···O4^i^	3.105 (4)	H12A···O4^iii^	2.8500
C8···O1	3.052 (3)	H12B···H11C	2.4000
C8···C21^i^	3.400 (4)	H15···O1^viii^	2.7900
C10···O1^iv^	3.150 (3)	H15···N3	2.9200
C10···C3	3.159 (4)	H15···C2^viii^	2.8500
C11···C6	3.058 (4)	H15···C22	3.0200
C13···O4	3.111 (4)	H15···H22A	2.5400
C15···C3^v^	3.593 (4)	H16···C4^viii^	3.0800
C15···C22	3.202 (4)	H16···C5^viii^	2.8500
C16···C4^v^	3.546 (4)	H16···C6^viii^	3.0500
C18···O1^vi^	3.355 (3)	H17···H10C^x^	2.5300
C18···C24	3.157 (4)	H18···C24	2.9500
C18···O2^vi^	3.329 (3)	H18···O1^vi^	2.6600
C20···O3	3.085 (3)	H18···O2^vi^	2.4900
C20···O2^vi^	3.322 (3)	H18···C1^vi^	3.0700
C21···C8^iv^	3.400 (4)	H21A···O3	2.3300
C22···C15	3.202 (4)	H21A···C12^iv^	3.0300
C22···O3^iv^	3.128 (3)	H21B···O1^viii^	2.8700
C22···O1^viii^	3.400 (4)	H21B···H22C	2.5300
C23···O2^vi^	3.329 (4)	H21C···O2^iv^	2.7600
C24···O2^vi^	3.224 (4)	H21C···C8^iv^	2.8200
C24···C18	3.157 (4)	H21C···H22B	2.5400
C1···H22C^ii^	2.8800	H22A···C14	2.4500
C1···H18^iii^	3.0700	H22A···C15	2.5600
C2···H15^ii^	2.8500	H22A···C24^iv^	2.9400
C2···H10A	2.4500	H22A···H15	2.5400
C3···H10A	2.6300	H22A···H24C^iv^	2.5000
C4···H16^ii^	3.0800	H22B···C11^xii^	3.0900
C5···H16^ii^	2.8500	H22B···H11B^xii^	2.4800
C6···H11B	2.9800	H22B···H21C	2.5400
C6···H16^ii^	3.0500	H22C···O1^viii^	2.8300
C6···H11A	2.6900	H22C···O3^iv^	2.6100
C7···H11A	2.5400	H22C···C1^viii^	2.8800
C8···H21C^i^	2.8200	H22C···H21B	2.5300
C10···H3	2.9800	H23A···O4	2.3800
C11···H22B^ix^	3.0900	H23B···O2^vi^	2.7100
C11···H6	2.7800	H23C···H5	2.4600
C11···H10A^i^	2.9500	H23C···H24C	2.2900
C12···H21A^i^	3.0300	H24A···C18	3.0800
C13···H10B^v^	2.9500	H24A···O2^vi^	2.5600
C13···H3^v^	3.0100	H24B···C18	2.7900
C14···H22A	2.4500	H24B···C19	2.5300
C14···H3^v^	2.8800	H24C···H22A^i^	2.5000
C15···H22A	2.5600	H24C···H23C	2.2900
			
C1—N1—C9	119.8 (2)	H12B—C12—H12C	109.00
C1—N1—C10	125.4 (2)	N2—C12—H12A	110.00
C9—N1—C10	114.7 (2)	N2—C12—H12B	109.00
C8—N2—C11	124.9 (2)	N2—C12—H12C	109.00
C8—N2—C12	119.6 (3)	H12A—C12—H12B	109.00
C11—N2—C12	115.6 (2)	H12A—C12—H12C	110.00
C13—N3—C22	124.7 (2)	O3—C13—N3	123.2 (3)
C21—N3—C22	115.2 (2)	O3—C13—C14	118.5 (2)
C13—N3—C21	120.0 (2)	N3—C13—C14	118.2 (2)
C23—N4—C24	116.3 (3)	C13—C14—C15	119.7 (2)
C20—N4—C23	118.4 (3)	C13—C14—C19	120.5 (2)
C20—N4—C24	124.2 (3)	C15—C14—C19	119.5 (3)
O1—C1—C2	118.6 (2)	C14—C15—C16	120.7 (3)
O1—C1—N1	123.9 (3)	C15—C16—C17	119.9 (3)
N1—C1—C2	117.5 (2)	C16—C17—C18	120.0 (3)
C1—C2—C3	119.7 (3)	C17—C18—C19	120.5 (2)
C1—C2—C7	120.2 (2)	C14—C19—C18	119.4 (3)
C3—C2—C7	119.8 (2)	C14—C19—C20	119.6 (2)
C2—C3—C4	120.4 (3)	C18—C19—C20	120.6 (2)
C3—C4—C5	120.0 (3)	O4—C20—N4	122.8 (3)
C4—C5—C6	120.1 (3)	O4—C20—C19	118.4 (3)
C5—C6—C7	120.2 (3)	N4—C20—C19	118.9 (3)
C6—C7—C8	121.0 (3)	C14—C15—H15	120.00
C2—C7—C6	119.4 (2)	C16—C15—H15	120.00
C2—C7—C8	119.3 (2)	C15—C16—H16	120.00
N2—C8—C7	118.2 (2)	C17—C16—H16	120.00
O2—C8—N2	123.3 (3)	C16—C17—H17	120.00
O2—C8—C7	118.5 (2)	C18—C17—H17	120.00
C2—C3—H3	120.00	C17—C18—H18	120.00
C4—C3—H3	120.00	C19—C18—H18	120.00
C3—C4—H4	120.00	N3—C21—H21A	109.00
C5—C4—H4	120.00	N3—C21—H21B	109.00
C6—C5—H5	120.00	N3—C21—H21C	109.00
C4—C5—H5	120.00	H21A—C21—H21B	110.00
C5—C6—H6	120.00	H21A—C21—H21C	109.00
C7—C6—H6	120.00	H21B—C21—H21C	110.00
N1—C9—H9B	109.00	N3—C22—H22A	109.00
N1—C9—H9A	109.00	N3—C22—H22B	109.00
H9A—C9—H9C	109.00	N3—C22—H22C	109.00
N1—C9—H9C	110.00	H22A—C22—H22B	109.00
H9A—C9—H9B	109.00	H22A—C22—H22C	109.00
H9B—C9—H9C	109.00	H22B—C22—H22C	109.00
N1—C10—H10C	109.00	N4—C23—H23A	109.00
N1—C10—H10A	109.00	N4—C23—H23B	109.00
N1—C10—H10B	109.00	N4—C23—H23C	109.00
H10A—C10—H10C	110.00	H23A—C23—H23B	110.00
H10B—C10—H10C	109.00	H23A—C23—H23C	109.00
H10A—C10—H10B	109.00	H23B—C23—H23C	110.00
H11A—C11—H11B	109.00	N4—C24—H24A	109.00
H11A—C11—H11C	109.00	N4—C24—H24B	109.00
N2—C11—H11A	109.00	N4—C24—H24C	109.00
N2—C11—H11B	110.00	H24A—C24—H24B	109.00
N2—C11—H11C	109.00	H24A—C24—H24C	109.00
H11B—C11—H11C	109.00	H24B—C24—H24C	109.00
			
C9—N1—C1—O1	3.8 (4)	C3—C4—C5—C6	−1.4 (4)
C10—N1—C1—O1	−173.5 (3)	C4—C5—C6—C7	0.7 (4)
C9—N1—C1—C2	−172.2 (2)	C5—C6—C7—C8	175.2 (3)
C10—N1—C1—C2	10.5 (4)	C5—C6—C7—C2	1.3 (4)
C12—N2—C8—C7	−173.4 (2)	C6—C7—C8—N2	55.4 (4)
C11—N2—C8—O2	−177.3 (3)	C2—C7—C8—O2	51.7 (4)
C12—N2—C8—O2	4.1 (4)	C2—C7—C8—N2	−130.7 (3)
C11—N2—C8—C7	5.2 (4)	C6—C7—C8—O2	−122.3 (3)
C21—N3—C13—O3	−6.4 (4)	O3—C13—C14—C19	−58.4 (4)
C22—N3—C13—O3	168.5 (3)	N3—C13—C14—C15	−60.6 (4)
C21—N3—C13—C14	169.5 (2)	N3—C13—C14—C19	125.6 (3)
C22—N3—C13—C14	−15.7 (4)	O3—C13—C14—C15	115.4 (3)
C23—N4—C20—O4	−8.3 (4)	C15—C14—C19—C18	1.0 (4)
C23—N4—C20—C19	171.2 (3)	C15—C14—C19—C20	174.1 (3)
C24—N4—C20—C19	3.6 (4)	C19—C14—C15—C16	−0.7 (4)
C24—N4—C20—O4	−175.9 (3)	C13—C14—C19—C18	174.9 (3)
O1—C1—C2—C3	−114.6 (3)	C13—C14—C15—C16	−174.6 (3)
O1—C1—C2—C7	59.1 (4)	C13—C14—C19—C20	−12.1 (4)
N1—C1—C2—C3	61.6 (4)	C14—C15—C16—C17	−0.2 (5)
N1—C1—C2—C7	−124.7 (3)	C15—C16—C17—C18	0.8 (5)
C7—C2—C3—C4	2.0 (4)	C16—C17—C18—C19	−0.4 (4)
C1—C2—C7—C6	−176.4 (3)	C17—C18—C19—C14	−0.5 (4)
C1—C2—C3—C4	175.8 (3)	C17—C18—C19—C20	−173.4 (3)
C1—C2—C7—C8	9.5 (4)	C14—C19—C20—N4	119.4 (3)
C3—C2—C7—C6	−2.7 (4)	C18—C19—C20—O4	111.9 (3)
C3—C2—C7—C8	−176.7 (3)	C18—C19—C20—N4	−67.7 (4)
C2—C3—C4—C5	0.0 (4)	C14—C19—C20—O4	−61.1 (4)

Symmetry codes: (i) *x*+1, *y*, *z*; (ii) *x*+1, *y*, *z*−1; (iii) −*x*+1, *y*+1/2, −*z*+1; (iv) *x*−1, *y*, *z*; (v) *x*, *y*, *z*+1; (vi) −*x*+1, *y*−1/2, −*z*+1; (vii) *x*, *y*, *z*−1; (viii) *x*−1, *y*, *z*+1; (ix) *x*+2, *y*, *z*; (x) −*x*, *y*−1/2, −*z*+1; (xi) −*x*, *y*+1/2, −*z*+1; (xii) *x*−2, *y*, *z*.

### Hydrogen-bond geometry (Å, º)

Cg1 is the centroid of the C2–C7 ring.

**Table:**

*D*—H···*A*	*D*—H	H···*A*	*D*···*A*	*D*—H···*A*
C3—H3···O3^vii^	0.93	2.60	3.518 (3)	170
C5—H5···O4^i^	0.93	2.58	3.141 (4)	119
C6—H6···O4^i^	0.93	2.51	3.105 (4)	122
C18—H18···O2^vi^	0.93	2.49	3.329 (3)	150
C24—H24*A*···O2^vi^	0.96	2.56	3.224 (4)	127
C16—H16···*Cg*1	0.93	2.97	3.810 (4)	124

Symmetry codes: (i) *x*+1, *y*, *z*; (vi) −*x*+1, *y*−1/2, −*z*+1; (vii) *x*, *y*, *z*−1.
